# *APOE* Gene Associated with Cholesterol-Related Traits in the Hispanic Population

**DOI:** 10.3390/genes12111768

**Published:** 2021-11-08

**Authors:** Stephanie Lozano, Victoria Padilla, Manuel Lee Avila, Mario Gil, Gladys Maestre, Kesheng Wang, Chun Xu

**Affiliations:** 1 Department of Science, Graduate College of Biochemistry and Molecular Biology, University of Texas Rio Grande Valley, Edinburg, TX 78539, USA; stephanie.lozano01@utrgv.edu; 2 Department of Health and Biomedical Science, College of Health Affairs, University of Texas Rio Grande Valley, Brownsville, TX 78520, USA; victoria.padilla01@utrgv.edu (V.P.); manuel.leeavila01@utrgv.edu (M.L.A.); 3 Department of Psychological Science, University of Texas Rio Grande Valley, Brownsville, TX 78520, USA; mario.gil@utrgv.edu; 4 Department of Neuroscience, School of Medicine, University of Texas Rio Grande Valley, Harlingen, TX 78539, USA; 5 Neuroscience and School of Medicine, University of Texas Rio Grande Valley, Edinburg, TX 78539, USA; gladys.maestre@utrgv.edu; 6 Health Sciences Center, Department of Family and Community Health, School of Nursing, West Virginia University, Morgantown, WV 26506, USA

**Keywords:** APOE, hypercholesteremia, cardiovascular diseases, dementia

## Abstract

Genetic variants in the apolipoprotein E (*APOE*) gene are associated with lipid metabolism and lipid-related traits in the non-Hispanic population. There have been limited studies regarding the association between the *APOE* gene and hypercholesterolemia in the Hispanic population; therefore, our aim for this study is to examine the APOE gene’s associations with cholesterol level and its related phenotypes. The *APOE* gene consists of three different alleles, ε2, ε3, and ε4, with ε4 being associated with dementia and cardiovascular diseases. A total of 1,382 subjects were collected from the Texas Alzheimer’s Research and Care Consortium (TARCC, N = 1320) and the Initial Study of Longevity and Dementia from the Rio Grande Valley (ISLD-RGV, N = 62). Questionnaires on demographics, medical history, and blood/saliva samples were collected and *APOE* genotypes were performed. We observed allele frequencies of the *APOE* ε3 (96.7%), ε4 (22.6%) and ε2 (6.8%) alleles, respectively. Multivariable logistic regression revealed a significant association between the *APOE* ε4 allele and hypercholesteremia (*p* = 1.8 × 10^−4^) in our studied Hispanic population. We prove for the first time, that the APOE ε4 allele increases the risk for hypercholesterol in Hispanics. Further research is needed to confirm and supports our current findings.

## 1. Introduction

Several genes have been suggested for abnormal lipid and/or lipid related phenotypes; among these candidate genes, the apolipoprotein E (APOE) gene is well documented [1,2]. The *APOE* genetic polymorphism involves the coding region of the *APOE* gene and results in alterations in the gene product, which affect the metabolic rate of the lipoprotein particles [3,4]. *APOE* is a multifunctional protein, which plays an important role in lipoprotein metabolism and has been shown to be associated with maintaining the homeostasis of cholesterol levels and transporting lipids [5]. The genetic polymorphism of the *APOE* gene is mainly attributable to three alleles, ε2, ε3, and ε4. According to a meta-analysis, the allele frequencies of the *APOE* gene were found to differ in different ethnic populations [6]; for example, the *APOE* ε4 allele is particularly enriched in the indigenous populations of Central Africa (40%), Oceania (37%), Africa American (31%), and Australia (26%), and Caribbean-Hispanic (22%), but less common in the Mediterranean area or South China (<10%) [7,8]. However, there is a lack of studies on *APOE* ε4 allele frequency and its association with cholesterol phenotypes in the U.S. Hispanic population. A recent study on *APOE* gene, protein function and interaction with dietary factors suggests that *APOE* ε3 is most prevalent worldwide, despite the local accumulation of *APOE* ε4 in indigenous populations, which may partly explain the different levels of beneficial and adverse effects in different ethnic backgrounds [9].

Accumulated studies have demonstrated that the *APOE* ε4 allele is associated with several health conditions, including cholesterol-related phenotypes reported in non-Hispanic populations. Another study has also demonstrated that total cholesterol level is higher in *APOE* ε4 allele carriers compared to those with the *APOE* ε2 allele in the Afro-Caribbean population [10]. It was also shown that the low-density lipoprotein cholesterol (LDL)-cholesterol level is lower in *APOE* ε2 carriers compared to individuals with homozygotes *APOE* ε3 and *APOE* ε4 alleles in the Afro-Caribbean population [10]. Another recent Meta-Analysis based on 27 studies with 3136 dementia and 3103 healthy controls reported that circulating cholesterol was significantly increased in *APOE* ε4 carriers in mixed populations [11]. The cholesterol-associated APOE ε4 allele was also reported in the Saudi—[12] White—[13,14,15,16], Afro-Caribbean—[10], and Asian—[17,18] populations. The role of *APOE* genetic variation on interindividual variation in the plasma cholesterol has been well-established, especially LDL-C in non-Hispanic White and African Black populations [19]. A recent genome-wide association study (GWAS) indicated that *APOE* ε3 allele, as a common allele, is also associated with high-density lipoprotein-cholesterol (HDL-C) and triglyceride (TG) levels in the White population [20]. Furthermore, a research study suggests a low frequency of *APOE* ε2 and ε4 alleles in the Hispanic population in comparison with non-Hispanic populations. Hispanic or Latino refers to a person of Cuban, Mexican, Puerto Rican, South or Central American, or other Spanish culture or origin regardless of race and is an admixed population [21,22]. The *APOE* ε4 allele is not associated with the risk of hypercholesterol in the U.S. Hispanic population.

Moreover, the complications of abnormal lipid levels (e.g., atherosclerosis, hypertension and/or diabetes, cardiovascular disease, CVD) were also observed to be associated with the *APOE* ε4 allele in the Asian population [23]. The *APOE* ε4 allele and the genotypes (ε3/ε4 and ε4/ε4) were associated with an increased risk of hypertension in different population studies, for example, in Asians and Caucasians [6]. Likewise, the individuals who carried the genotypes *APOE* ε3/ε4 and ε4/ε4, had high levels of LDL-C and increased blood pressure [6]. To date, only one study in the Mexican population shows that the *APOE* ε4 allele was associated with the lowest levels of HDL-C [24]. However, to date, there is no study of the *APOE* gene in association with cholesterol-related phenotypes in the U.S. Hispanic population.

Studies on the plasma lipid composition have shown differences between non-Hispanic Whites and Hispanics; Hispanics have higher circulating TG and lower HDL cholesterol levels [25]. Also, a systemic review reported the higher levels of C-reactive protein (CPR) in Hispanics than Caucasians due to differences in social economic situation and racial/ethnicity [25,26].

Moreover, over 6 decades ago, a relationship between thyroid autoimmunity and hyperlipidemia was suggested [27]. Therefore, we also tested the association between thyroid-related phenotypes and hypercholesteremia in the U.S. Hispanic population.

The *APOE* ε4 allele is associated with lipid-related traits or diseases mainly reported in non-Hispanic populations, which had never been explored in the Hispanic population. Thus, the current study investigated the frequencies of the *APOE* alleles and studied their relationship with cholesterol levels and lipid-related traits in the Hispanic population in the current study.

## 2. Materials and Methods

This study is an analysis of data collected by the Texas Alzheimer’s Research and Care Consortium TARCC (N = 1320) in combination with our data, Initial Study of Longevity and Dementia from the Rio Grande Valley (ISLD-RGV, N = 62), resulting in a total of 1382 subjects. The study protocols were approved by the corresponding Institutional Ethics Committees and Institutional Review Boards. A written consent form was obtained from each participant or by their legally authorized proxies, and these were gathered before data collection began as in previous studies [28,29].

The first group of data were from ISLD-RGV. The Hispanic control subjects were matched to cases based on age, gender, and ethnicity. Patients with hypercholesterolemia, diabetes, other chronic disorders, and healthy subjects were recruited from the RGV, which included the regions of Brownsville and McAllen, Texas. Participants were recruited from local adult daycare centers and communities. In addition, questionnaires on lifestyle (several questions [30] and medical history) modified based on [31] were also collected during the interview.

The second group of data were collected from the TARCC study, which is a well-characterized, ethnically diverse sample with annual longitudinal follow-up, described in detail in previous reports [32,33]. The participants underwent a standardized annual examination, which included a medical evaluation, neuropsychological testing, and clinical interview.

Phenotypes of demographics, medical history and diseases were assigned no more than three values, denoted by 0, and 1. For example, to specify the presence or absence of phenotypes (e.g., hypercholesterolemia, diabetes, and hypertension), the values 0 (Absent) and 1 (Present) were given. All diagnoses were based on either self-report and/or standardized enzymatic procedures. Hypercholesterolemia was defined as follows: 0 = Absent, and 1 = Inactive/Active. Serum cholesterol > 220 mg/dL was defined as hypercholesterolemia. Hypertension was defined as: 0 = Absent, 1 = Inactive/Active.

### 2.1. DNA Isolation and Genotyping

Genetic data were collected to study genetic determinants, such as *APOE* genotypes and alleles. DNA extraction was performed using two different methods: 1320 blood samples were collected from TARCC, and 62 saliva samples were collected from ISLD-RGV. DNA isolation and genotyping of the TARCC’s samples were described in detail in previous studies [33,34]. For the collection of ISLD-RGV saliva samples, those who consented to the study were asked to provide 2 mL of saliva. The samples were collected using Oragene DISCOVER (OGR-500) (DNA Genotek. Ottawa, ON, Canada), a self-collecting kit, from the company DNA Genotek Collection and collected according to OGR-500 protocols [35]. The saliva samples were stored at a temperature of 4 °C until DNA extraction. DNA isolation from saliva was performed following the standardized laboratory protocol described by DNA Genotek using PrepIT^®^•L2P (DNA Genotek, Ottawa, ON, Canada).

The genotype for the *APOE* gene was determined by two separate methods. TaqMan single-nucleotide polymorphism (SNPs) assays were used to analyze the SNPs, and rs7412 and rs429358 were used to identify the three main *APOE* alleles (ε3, ε2, and ε4) for the ISLD-RGV sample. TARCC utilized the Affymetrix Genome-Wide Human SNP Array 6.0 to collect SNPs rs7412 and rs429358 data, as in previous studies [36]. The validity of the recorded genotypes was verified by executing GWAS and a whole exome sequence (WES) to compare replicated samples of the same pipeline [37]. SNPs data were identified as either an ε2−, ε3−, ε4− (the minus sign states that the allele is absent) or ε2+, ε3+, ε4+ (the plus sign “+” indicates that the allele presents), participants who carried one copy of the allele, being heterozygous or two alleles, homozygous). From a total of 1382 participants, only 1320 participants had *APOE* genotypes. 

### 2.2. Statistical Analyses

Statistical analysis was performed according to *APOE* ε2, ε3 and ε4 carriers (ε2+, ε3+, and ε4+) and non-carriers (ε2−, ε3−, and ε4−). In total, several variables (e.g., BMI, age, and education) were used to generate statistical reports from the studied population. The Statistical Package for Social Sciences (SPSS) version 26 was used to perform statistical analysis. A two-tailed *t*-test and chi-square analysis were used to examine the role of each potential risk factor for cholesterol related phenotypes; then multivariable logistic regression was used to adjust for all the potential risk factors (e.g., sex, age, and education). To test whether the *APOE* alleles were independently associated with several phenotypes (e.g., cholesterol-related traits) in the subjects, logistic regression models were constructed. Differences with two-tailed probability values of *p* < 0.05 were considered to be statistically significant. The odds ratio (OR) was used to determine the strength of associations between alleles and phenotypes. 

### 2.3. Power Analyses 

As in our previous studies, including [38], a power analysis was estimated for known genes using a case-control study design for discrete traits using Genetic Power Calculator [39] (https://zzz.bwh.harvard.edu/gpc/ (accessed on 1 November 2021)). The power to detect the association between *APOE* alleles and phenotypes was estimated as 94%, based on the total sample of 1333 subjects, including 860 affected subjects with disease phenotypes (e.g., hypercholesterolemia), 460 matched control, and marker allele frequency of 0.2 since these allele frequencies are the minor allele frequencies of the tested *APOE* gene.

## 3. Results

### 3.1. Descriptive Statistics of Hypercholesterolemia

A total of 1320 out of 1382 participants had *APOE* genotype data. The status of the 1320 participants was classified as 860 (65.2%) participants with high cholesterol levels, while 460 (34.8%) represented the control group in the present study. The demographic characteristics of hypercholesterolemia and non-hypercholesterolemia are shown in Table 1. A total of 589 females and 271 males had hypercholesterolemia. The mean age of hypercholesterolemia cases was 70.41 ± 9.12 years, which was found to be statistically significantly older than that in the control subjects (67.20 ± 9.85, *p* = 3.98 × 10^−9^, Table 1). The frequencies of hypertension, thyroid disease, and diabetes were statistically significantly higher in the patients with hypercholesterolemia as compared with the control group (*p* < 0.002). There was no difference in body mass index (BMI) between hypercholesterolemia (BMI = 30.35 ± 9.12) and the control group (BMI = 30.37 ± 7.23). 

### 3.2. Distributions of APOE Alleles

The study included 1382 Hispanic participants collected from the combined TARCC and ISLD-RGV data. A total of 961 (69.8%) subjects were females and 421 (30.2%) subjects were males. The allele frequencies of the *APOE* ε3 (96.7%), ε4 (22.6%) and ε2 (6.8%) alleles were observed (Table 2), which are similar to the frequencies in the Hispanic population in previous reports [36,37]. *APOE* ε4 allele status on demographics and cholesterol-related phenotypes is displayed in Table 3. In terms of the *APOE* ε4 allele, a total of 20.6% females, and 22.1% males carried at least one *APOE* ε4 allele (Table 3). A total of 211 (25.9%) participants with hypercholesterolemia carried at least one copy of the *APOE* ε4 allele. Chi-square test revealed a significant association between *APOE* ε4 allele and hypercholesterolemia (*p* = 1.17 × 10^−4^).

### 3.3. Multivariable Logistic Analysis

A multivariable logistic regression analysis was performed, and the results further supported the association between the *APOE*-ε4 allele and hypercholesteremia (Table 4). After controlling for potential confounding factors (e.g., gender, education and *APOE* ε allele), we observed a statistically significant association between the *APOE* ε4 allele and hypercholesteremia (*p* = 1.8 × 10^−4^). The odds of having hypercholesteremia were 1.9-fold higher (95% CI, 1.36–2.67), in persons with an *APOE*-ε4 allele compared with those without. The *APOE* ε3 allele was not associated with hypercholesteremia (*p* = 0.484).

## 4. Discussion

This study demonstrates the *APOE* ε4 allele’s association with cholesterol in the Hispanic population, after controlling for covariates, such as gender, BMI, *APOE* ε3 allele, and education. This finding adds additional evidence to the association between the *APOE* ε4 allele with hypercholesterolemia. Increasing studies suggest an association between *APOE* ε4 allele and hypercholesterolemia in non-Hispanic populations. In this study, the Hispanic participants are 96.7% *APOE* ε3 allele carriers, followed by *APOE* ε4 allele (22.6%) and, lastly, the *APOE* ε2 allele (6.8%), which are comparable to the results of previous studies in Hispanics [40,41]. In our study, the hypercholesterolemia associated *APOE* ε4 allele suggests that individuals carrying the *APOE* ε4 allele(s) are at an increased risk of hypercholesterolemia development in the Hispanic population. Previous studies have also marked the *APOE* ε4 allele as a determinant for hypercholesterolemia in the Algerian population [42] and other non-Hispanic populations. A case-control study showed that participants residing in Valencia, Spain, that were *APOE* ε4 allele carriers had a higher risk of hypercholesterolemia than those that were not *APOE* ε4 allele carriers [43]. However, there were no associations between the *APOE* ε4 allele and obesity and hypertension in the our current study, which is consistent with the findings in previous reports in Mexican and European populations [24,44,45].

This is the first report of significant findings of the *APOE* ε4 allele’s association with hypercholesterolemia in the Hispanic population. A previous study of 425 Hispanic participants (193 with and 232 without cerebral amyloid angiopathy) reported a higher prevalence of stroke and hypercholesterolemia in non-*APOE* ε4 allele carriers [45]. However, there have not been more studies up to date involving Hypercholesterolemia, the *APOE* ε4 allele, and the Hispanic population. A study with a much bigger sample size of 1997 Mexican Amerindians (MA) showed an association between those MA who carried the *APOE* ε4 allele and a low level of HDL-C and higher levels of LDL-C [24], which partially supports our current findings. However, we focused on total cholesterol level and patients with hypercholesterolemia. Another study from the Kashmiri population shows that patients with CAD carrying the ε4 allele had significantly higher total cholesterol (TC) and LDL levels [46].

Regarding the statistical analysis of *APOE* ε3 allele, our study demonstrated no significant associations between the *APOE* ε3 allele and obesity, hypercholesterolemia, or CVD in our Hispanic population. Furthermore, it has been suggested that the *APOE* ε2 allele is considered a protective allele against CVD by lowering the levels of LDL [47,48].

In addition, cholesterol levels have been shown to regulate inflammatory and immune processes, as shown in Chinese population [49] but little is known about their association with thyroid disease in the Hispanic population. Based on the current study, we observed that 73.3% of patients with hyperlipidemia are also comorbid with thyroid disease (Table 1).

### Strength and Limitations

The strengths of the current study are as follows: (1) additional evidence of 22% of *APOE* ε4 allele was observed, and (2) this is the first study showing that the *APOE* ε4 allele increases the risk of hypercholesterol in the U.S. Hispanic population. The current results can be used for future confirmation in a meta-analysis of this unique population. The limitations of our study consist of (1) the sample size, comprising of a total of 1382 participants, was moderate or small in our study, although we showed a high statistical power of 94%, after further dividing the subjects, for an example based on subjects with and without the *APOE* ε4 allele. This small sample size may decrease the statistical power and lead to a type II error [18,44], so a larger sample size is required to obtain more reliable results. The current study is ongoing, we will continue to recruit subjects to increase statistical power and conduct a more specific analysis, such as associations between *APOE* ε4 allele or its genotypes (ε4/ε4, ε3 ε4 or ε3/ε2, etc) and several cholesterol related phenotypes (e.g., hypertension, thyroid disease). (2) Our studied population, the Hispanics, is very diverse and is poorly understood. Studies have observed that this ethnicity is diverse in genetic ancestry, culture, and environmental exposures [50]. More studies should be made, with a larger sample size, to further prove the associations found in this study. (3) Phenotypic heterogeneity was observed in our studied samples; for example, patients with hypercholesterolemia may have comorbidities such as diabetes or hypertension. (4) Total cholesterol was tested, which may have more phenotypic heterogeneity in the current study. Therefore, studies on more specific type(s) of cholesterol such as HDL-cholesterol and LDL-cholesterol, in association with *APOE* alleles, are needed in the future. (5) Cholesterol metabolism among different *APOE* genotypes (e.g., ε4/ ε4, ε3/ε 4) and sex differences in the effects of *APOE* alleles along the *APOE* pathway were not tested because reducing the sample size would result in a loss in statistical power. This could be a future study direction.

## 5. Conclusions

The current study emphasizes the prevalence of high cholesterol levels and the effects that this may have on genetics (e.g., *APOE* gene) and demographics (e.g., sex, age, and education) in the Hispanic population. The results of the current study demonstrate that the *APOE* e4 allele carries a high risk for cholesterol-related traits, including hypercholesterolemia, in the Hispanic population. No associations were found between the *APOE* ε3 or *APOE* ε2 allele and cholesterol-related traits in the current population. While this study aims to aid doctors in the provision future care plans to detect and prevent metabolic syndrome symptoms before it is too late, further studies are needed to confirm our current findings in this population. This work functions to highlight how the understudied Hispanic population can further our understanding of the genetics of cholesterol-related phenotypes.

## Figures and Tables

**Table 1:** Results of chi-square^2^ analysis and *t*-test for demographic and health characteristics in hypercholesterolemia.

	Controls (N = 460)	Hypercholesterolemia Cases (N = 860)	*p* Value
Age (mean ± SD)	67.20 ± 9.85	70.41 ± 9.12	3.98 × 10^−9^
Males (N, %) N = 399	128 (32.08%)	271 (67.92%)	0.165
Females (N, %) N = 921	332 (36.05%)	589 (63.95%)	
BMI ^a^ (mean ± SD)	30.37 ± 7.23	30.35 ± 6.24	0.956
Hypertension (N, %) N = 874 Non-Hypertension (N, %) N = 446	231 (26.43%) 229 (51.3%)	643 (73.57%) 217 (48.7%)	<0.00001
Thyroid (N, %) N = 273 Non-Thyroid (N, %) N = 1047	73 (26.74%) 387 (37%)	200 (73.26%) 660 (63.0%)	0.002
Diabetes (N, %) N = 489 Non-Diabetes (N, %) N = 831	93 (19.02%) 367 (44.2%)	396 (80.98%) 464 (55.8)	<0.00001
Education (mean ± SD)	9.74 ± 4.66	10.45 ± 4.62	0.518

*p* value, *t*-test for continuous variables and chi-square test for categorical variables. ^a^ BMI—body mass index. Numerical values are expressed as the mean ± SD.

**Table 2:** The distributions of *APOE* alleles in the studied Hispanic population.

*APOE* ε3+	*APOE* ε4+	*APOE* ε2+
96.7%	22.6%	6.8%

*APOE* ε3+, ε4+, or ε2+: carrying at least one copy of *APOE* alleles.

**Table 3:** *APOE* **ε**4 allele status on demographics and cholesterol related phenotypes in our studied population.

	*APOE* ε4 − ^a^ (N = 1042)	*APOE* ε4 + ^b^ (N = 278)	*p* Value
Age (mean ± SD)	69.33 ± 9.40	70.75 ± 9.45	0.027
Females (N, %) N = 921	731 (79.37%)	190 (20.63%)	0.624
Males (N, %) N = 399	311 (77.94%)	88 (22.06%)	
Education (mean ± SD)	10.17 ± 4.62	10.41 ± 4.74	0.236
^c^ BMI (mean ± SD)	30.44 ± 6.62	29.61 ± 6.35	0.064
Hypercholesterolemia (N, %)	605 (74.14%)	211 (25.86%)	1.17 × 10^−4^
Hypertension (N, %) N = 832	640 (76.92%)	192 (23.08%)	0.564
Diabetes (N, %) N = 459	365 (79.52%)	94 (20.48%)	0.170
Obese (N, %) N = 112	88 (78.57%)	24 (21.43%)	0.639

*p* value, *t* test for continuous variables and Chi-square test for categorical variables. ^a^ The *APOE* ε4 allele is absent (*APOE* ε4-). ^b^ Carrying at least one copy of *APOE* ε4 (*APOE* ε4+). ^c^ BMI- body mass index. Numerical values are expressed as the mean ± SD.

**Table 4:** Logistic Regression Analysis of Cholesterol-Related Phenotypes.

	Hypercholesterolemia Group (N = 860) vs. Control Group (N = 460)	
	OR ^a^ (95%CI ^b^)	*p* Value
Sex	0.86 (0.65, 1.13)	0.28
BMI ^c^	1.00 (0.99, 1.02)	0.53
*APOE* ε3+	1.34 (0.59, 3.06)	0.484
*APOE* ε4+	1.90 (1.36, 2.67)	1.8 × 10^−^^4^
Education	0.94 (0.75, 1.19)	0.61

^a^ OR—odds ratio, ^b^ CI—confidence interval. ^c^ BMI— body mass index. Carrying at least one copy of *APOE* ε4 (*APOE* ε4+). Carrying at least one copy of *APOE* ε3 (APOE ε3+).

## Data Availability

Part of date was from the Texas Alzheimer’s Research and Care Consortium (TARCC) at https://www.txalzresearch.org/, accessed on 28 May 2020).
